# A Gene Co-Expression Network in Whole Blood of Schizophrenia Patients Is Independent of Antipsychotic-Use and Enriched for Brain-Expressed Genes

**DOI:** 10.1371/journal.pone.0039498

**Published:** 2012-06-27

**Authors:** Simone de Jong, Marco P. M. Boks, Tova F. Fuller, Eric Strengman, Esther Janson, Carolien G. F. de Kovel, Anil P. S. Ori, Nancy Vi, Flip Mulder, Jan Dirk Blom, Birte Glenthøj, Chris D. Schubart, Wiepke Cahn, René S. Kahn, Steve Horvath, Roel A. Ophoff

**Affiliations:** 1 Department of Medical Genetics, University Medical Center Utrecht, Utrecht, The Netherlands; 2 Department of Psychiatry, Rudolf Magnus Institute of Neuroscience, University Medical Center Utrecht, Utrecht, The Netherlands; 3 Julius Centre for Health Sciences and Primary Care, University Medical Center Utrecht, Utrecht, The Netherlands; 4 Department of Human Genetics, David Geffen School of Medicine, University of California Los Angeles, Los Angeles, California, United States of America; 5 Center for Neurobehavioral Genetics, Semel Institute for Neuroscience & Human Behavior, University of California Los Angeles, Los Angeles, California, United States of America; 6 Parnassia Bravo Group, The Hague, The Netherlands; 7 Department of Psychiatry, University of Groningen, Groningen, The Netherlands; 8 Center for Clinical Intervention and Neuropsychiatric Schizophrenia Research, Psychiatric University Center Glostrup, University of Copenhagen, Glostrup, Denmark; 9 Department of Biostatistics, School of Public Health, University of California Los Angeles, Los Angeles, California, United States of America; Catholic University of Sacred Heart of Rome, Italy

## Abstract

Despite large-scale genome-wide association studies (GWAS), the underlying genes for schizophrenia are largely unknown. Additional approaches are therefore required to identify the genetic background of this disorder. Here we report findings from a large gene expression study in peripheral blood of schizophrenia patients and controls. We applied a systems biology approach to genome-wide expression data from whole blood of 92 medicated and 29 antipsychotic-free schizophrenia patients and 118 healthy controls. We show that gene expression profiling in whole blood can identify twelve large gene co-expression modules associated with schizophrenia. Several of these disease related modules are likely to reflect expression changes due to antipsychotic medication. However, two of the disease modules could be replicated in an independent second data set involving antipsychotic-free patients and controls. One of these robustly defined disease modules is significantly enriched with brain-expressed genes and with genetic variants that were implicated in a GWAS study, which could imply a causal role in schizophrenia etiology. The most highly connected intramodular hub gene in this module (*ABCF1)*, is located in, and regulated by the major histocompatibility (MHC) complex, which is intriguing in light of the fact that common allelic variants from the MHC region have been implicated in schizophrenia. This suggests that the MHC increases schizophrenia susceptibility via altered gene expression of regulatory genes in this network.

## Introduction

Schizophrenia is a severe mental disorder, affecting about 1% of the population worldwide. Heritability is estimated to be around 80%, but the underlying genes are largely unknown [1]. Large-scale, genome-wide studies have identified rare genomic microdeletions as well as common variants associated with the disease [2], [3], [4]. In a recent study, Purcell and colleagues demonstrated that data from genome-wide association studies (GWAS) for schizophrenia are compatible with a very large number of loci with common alleles (N>3,000), each with very small odds ratios (<1.05) contributing to disease susceptibility [5]. Additional approaches may be necessary to decipher the genetic basis of schizophrenia and related disorders [6]. Gene expression is an intermediate phenotype between gene sequence and complex traits and can therefore facilitate the study of genetic variation and disease susceptibility [7]. Genome-wide gene expression profiling has been used to aid in the discovery of genes involved in human diseases and to discriminate between disease subtypes [8].

Until now, gene expression studies of schizophrenia have been limited in size and have primarily made use of post-mortem brain tissue. Although these studies yielded interesting findings and highlighted genes involved in several biological pathways [9], [10], [11], [12], [13], [14], [15] no consistent biological pattern has emerged. This may be due to the limitation in sample size of each study as well as confounders that are related to RNA quality of post-mortem material, such as, post-mortem interval and pH [13]. For diagnostic purposes, it would be desirable to develop blood-based biomarkers for schizophrenia since whole blood collection is non-invasive and can be efficiently performed for relatively large cohorts of patients and controls even in early stages of disease manifestation. Although gene expression in whole blood is only moderately correlated with gene expression in brain tissue [16], [17], [18], [19] several studies suggest that gene expression in blood could serve as a marker of brain-related disease states, including schizophrenia [17], [18], [20], [21], [22], [23], [24].

Our study aims to identify genetic mechanisms underlying schizophrenia using blood-based gene expression profiles. We obtained genome-wide expression data from 121 schizophrenia patients (with 92 medicated and 29 antipsychotic-free subjects) and 118 healthy control subjects. Using a co-expression network-based approach, we identify a schizophrenia-associated cluster of co-expressed genes that was independent of medication-effects. Further analysis showed that this cluster is enriched with brain-expressed genes and that the hub gene is located in and regulated by the MHC-complex. Our results complement previous GWAS findings and may highlight new causal candidate genes for schizophrenia.

## Results

### Data Preprocessing

Gene expression data were transformed, normalized and filtered as described in the methods section. Three datasets can be distinguished and are described in Table 1. Dataset 1 includes schizophrenia patients on antipsychotics (n = 92) and healthy controls (n = 78). Dataset 2 consists of n = 29 antipsychotic-free schizophrenia patients and n = 40 healthy controls. This dataset was used to replicate the results obtained with dataset 1.

**Table 1:** Description of datasets.

	1: Schizophrenia dataset		2: Antipsychotic-free dataset	
	Controls	Cases	Controls	Cases
**Total**	78	92	40	29
**Mean age**	41 yrs	41 yrs	30 yrs	31 yrs
**Gender**	31M, 47F	66M, 26F	27M, 13F	21M, 8F
**Batch 1**			22	15
**Batch 2**	78	92	18	14
Country*	22 DK, 56 NL	92 NL	6 DK, 34 NL	6 DK, 23 NL
**Expression Array**	Illumina H-12 (16,707 genes)		Illumina H-8 & H-12 (12,704 genes)	

For this study, three datasets were used; schizophrenia cases and controls, an antipsychotic-free set and a control dataset. Age and gender information is given for cases and controls separately. Gene expression data was generated in two batches (batch 1: Illumina H-8 and batch 2: Illumina H-12) and collected at different sites, information given in the fourth and fifth row). The batch effect resulting from the use of different arrays on different time points in the latter set was removed using the SampleNetwork R package [62]. The number of expressed genes is given in the last row. *DK  =  Denmark and NL  =  The Netherlands.

### Network Reconstruction in Schizophrenia Cases and Controls

#### Network reconstruction

**Figure 1:**
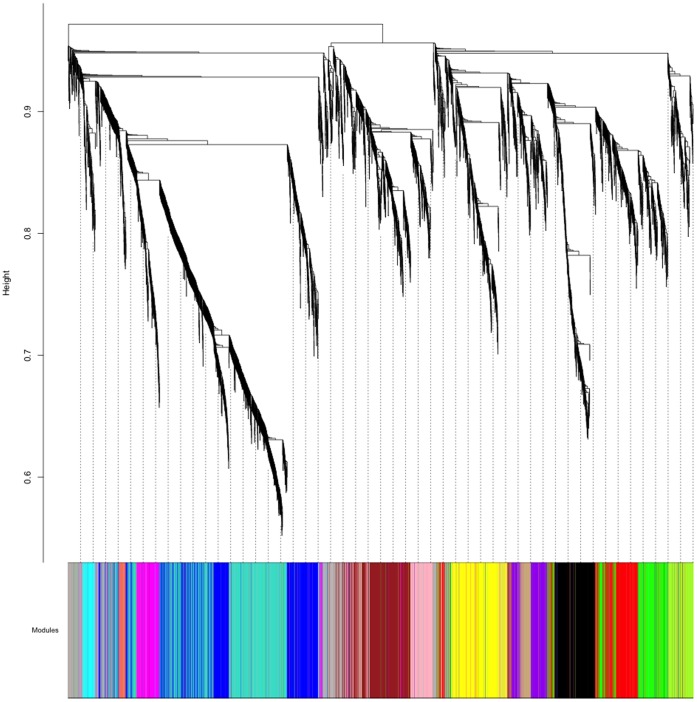
Network construction identifies distinct modules of co-expressed genes. The network was constructed using gene expression data of 92 medicated schizophrenia cases and 78 controls (dataset 1). The dendrogram was produced by average linkage hierarchical clustering of genes using 1-topological overlap as dissimilarity measure (see methods section). Modules of co-expressed genes were assigned colors corresponding to the branches indicated by the horizontal bar beneath the dendrogram.

A weighted gene co-expression network was constructed using dataset 1, independent of disease status, as described in the Methods section. For this, the 5,000 most variable transcripts were selected. The variance of the expression of these genes ranges from *HLA-A29.1* (average expression  = 12.10, variance  = 5.60) to *MSLN* (average expression  = 7.46, variance  = 0.04). Application of WGCNA procedure to genome-wide data (16,707 genes) results in almost identical findings (Table S1). The selection of the 5,000 most variable transcripts was used in a hierarchical clustering procedure to identify groups of co-expressed genes, termed ‘modules’. These correspond to the branches of the resulting clustering tree. Each module is assigned a unique color label, which is visualized in the color band underneath the cluster tree (Figure 1). We identified 14 modules ranging in size from 115 genes in the Cyan module to 789 in the Turquoise module. We also defined an improper (grey) module with genes not belonging to any of the 14 proper modules. The expression profiles of transcripts inside a given module were summarized by their first principal component (referred to as module eigengene). The module eigengene is a weighted (quantitative) average of the module gene expression profiles.

#### Co-expression modules related to schizophrenia status

To identify modules related to schizophrenia disease status, we regressed each of the 14 module eigengenes on disease status. Strikingly, 12 out of the 14 modules were significantly (FDR corrected *p*<0.05) associated with schizophrenia status in dataset 1 (Table 2). Of these 12 associated modules, the Green, Tan, Red, Yellow, Black and Pink modules were negatively correlated with schizophrenia, meaning that the genes in these modules are predominantly under-expressed in schizophrenia cases. In contrast, the Magenta, Turquoise, Salmon, Blue, Cyan and Greenyellow modules were positively correlated with disease status, containing mostly genes over-expressed in schizophrenia.

**Table 2:** Module eigengene significance for co-expression modules.

		Schizophrenia dataset		Antipsychotic-free dataset		
WGCNA Modules	# genes	t	Adjusted p-value	t	Adjusted p-value	Expressed in brain
Green	367	−6.26	3.8×10^−10^	−0.99	4.8×10^−01^	-
Magenta	226	5.51	3.5×10^−08^	−0.24	9.6×10^−01^	-
**Tan**	**129**	−**4.92**	**8.8**×**10** ^−**07**^	−**2.61**	**4.8**×**10** ^−02^	**61%**
Red	344	−4.63	3.6×10^−06^	−1.60	2.2×10^−01^	-
Turquoise	789	4.37	1.3 x10^−05^	1.97	1.2×10^−01^	-
Yellow	399	−3.82	1.3×10^−04^	−1.26	3.6×10^−01^	-
Salmon	121	3.02	2.5×10^−03^	2.51	4.8×10^−02^	52%
Blue	610	2.95	3.2×10^−03^	2.04	1.2×10^−01^	-
Cyan	115	2.87	4.1×10^−03^	−0.13	9.6×10^−01^	-
Greenyellow	197	2.59	9.7×10^−03^	−2.51	4.8×10^−02^	-
Black	321	−2.09	3.6×10^−02^	0.33	9.6×10^−01^	-
Pink	290	−2.03	4.2×10^−02^	−0.05	9.6×10^−01^	-
Purple	205	−1.07	2.9×10^−01^	-	-	-
Brown	447	−0.24	8.1×10^−01^	-	-	-

The modules that were found by WGCNA in the first dataset are listed together with the number of genes they contain (shown in the second column). Differences in cases and controls were tested using a linear model with FDR correction. Results for the medicated cases versus controls are presented in column three and four. The modules that were found to be differentially expressed were also tested for significance between cases and controls in the antipsychotic-free set, and results are presented in the fifth and sixth column. The last column indicates the percentage of module content that was also found to be expressed in brain (log_2_>4). For all genes in the other modules, this was found to be 45%. For the Tan module, this was significantly higher (Fisher *p* = 4.3×10^−4^).

### Replication of Co-expression Network Results in the Antipsychotic-free Dataset

#### Network preservation in the antipsychotic-free dataset

Based on our data, we hypothesized that the large-scale differential gene expression observed in schizophrenia patients might be caused by antipsychotic drug treatment. To test this hypothesis and to eliminate possible confounding effect of antipsychotic medication on gene expression profiles, a second dataset of antipsychotic-free schizophrenia patients (n = 29) and unaffected controls (n = 40) was used to replicate the results (see Table 1). Of the 5,000 genes used to construct the previous network, 4,464 (89%) were also present in dataset 2, representing the overlap between the Illumina H-8 and H-12 arrays. WGCNA was performed for this dataset separately, using the module assignment (color codes) of the previous dataset. To quantitatively assess whether a specific module is preserved, we used a module preservation Z statistic implemented in the modulePreservation function of the WGCNA R library [25], [26]. A Z-statistic value larger than 2 indicates that the corresponding module is significantly preserved in the second set. We find that all of our modules have a preservation Z score larger than 8, which shows that they are highly preserved between the discovery sample of schizophrenia cases/controls and the antipsychotic-free dataset (Figure S1).

#### Gene co-expression modules related to schizophrenia status in antipsychotic-free set

The module eigengenes of the twelve modules that showed significant disease associations in the discovery dataset were examined in the antipsychotic-free data set using a regression analysis with a FDR of 5%. For only two (Tan and Salmon) out of 12 schizophrenia related modules could the disease association be replicated (*p*<0.05) in the second, antipsychotic-free, dataset. The Greenyellow module is also significantly associated with disease status in dataset 2 but in the opposite direction, i.e. its disease association cannot be replicated. Results are summarized in Table 2.

### Enrichment for Blood Cell Type Markers and Brain-expressed Genes

#### Module content reflects blood cell types

A previous study identified genes of which expression was correlated to major peripheral blood cell types (neutrophil count (51 genes), lymphocyte count (56 genes) and red blood cell size (56 genes)) [27]. These lists of genes were taken as input for the userListEnrichment function incorporated in the WGCNA package [28] that compares the number of overlapping genes to the maximal possible overlap. We find both the Black (11/35 of genes overlap; 31%, *p* = 5.6×10^−4^) and the Brown (9/35 genes overlap; 26% *p* = 6.9×10^−4^) module to be enriched for markers of the most prevalent leukocytes: neutrophils. The Red module was enriched for lymphocyte markers (10/37 genes overlap; 27%, *p* = 2.3×10^−5^) and the Yellow module for red blood cell size markers (22/29 genes overlap; 76%, *p* = 9.3×10^−21^). Finally, Whitney and colleagues [27] defined a set of 30 genes correlated with time of blood draw, that were overrepresented in our Cyan module (5/15 genes overlap; 33%, *p* = 5.5×10^−7^). No significant enrichment with blood cell markers could be found for the two validated disease modules (Tan and Salmon).

#### Enrichment of brain-expressed genes in disease-associated module

Next, we examined whether the two validated schizophrenia modules (Tan and Salmon) were enriched for brain-expressed genes using a publically available brain expression dataset [29]. Table 2 shows that the Tan module contains the highest number of genes expressed in brain (61%), followed by the Salmon module (52%). In comparison, 45% of the genes in the 10 non-significant modules were expressed in brain. When formally tested, the Tan module was the only module that was significantly enriched with brain-expressed genes (Fisher *p* = 4.3×10^−4^). Using the userListEnrichment function, we did not find the Tan module as a whole, or the subset of brain-expressed genes, to be enriched for markers of particular neuronal cell types (astrocytes, oligodendrocytes or neurons) [28], [30].

### Inner Module Structure and Genetic Control of the Tan Schizophrenia Module

**Figure 2:**
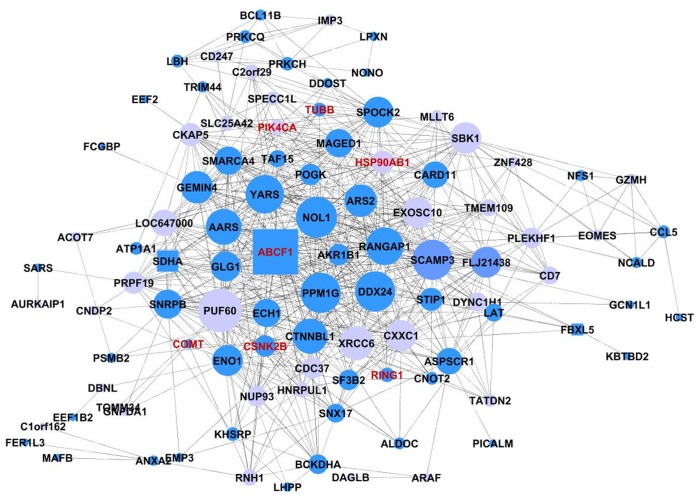
Visual representation of connections of genes in the Tan schizophrenia module. This figure shows target genes of the probes in the Tan schizophrenia module with the strongest connections only (*r* >0.64). Blue-colored nodes represent brain-expressed genes. Square-shape nodes indicate *cis*-regulation. Node size is related to the number of connections of that particular gene; a highly connected gene (i.e. ‘hub gene’) is therefore larger than genes with fewer connections. Red text indicates genes previously implicated in schizophrenia. Image created using Cytoscape software [69].

To determine genes that are centrally located in the Tan module, we calculated the intramodular connectivity. The intramodular connectivity (*k*-within) was calculated for each gene by summing the connection strengths with other module genes and dividing this number by the maximum intramodular connectivity. Genes with high intramodular connectivity are informally referred to as intramodular hub genes. The *k*-within statistics for the genes in the Tan module are presented in Table S2. In the Tan module, *ABCF1* (ATP-binding cassette, sub-family F (*GCN20*), member 1) on chromosome 6p is the most connected gene. We consulted a publically available human expression quantitative trait loci (eQTL) database generated on lymphoblastoid cells [31]. In the Tan module we find *SLC2A6*, *SDHA*, *DHRS1*, *sep-06*, *CNDP2*, *SIGIRR*, *FBXL5*, *DHX58* and the hub gene *ABCF1* to be in the list of heritable *cis*-regulated genes (LOD score and heritability estimates for genes in the Tan module from Dixon and colleagues [31] are given in Table S2). A visual representation of the Tan inner module structure and content is given in Figure 2.

### Gene Ontology Analysis of the Tan Schizophrenia Module

Ontology was assessed using Ingenuity (Ingenuity® Systems, www.ingenuity.com). Categories were considered significantly enriched when *p*<0.001. Top enriched categories for the Tan module include Cellular Development (smallest *p*-value = 4.2×10^−5^), Hematological System Development and Function (smallest *p* = 4.2×10^−5^), Hematopoiesis (smallest *p* = 4.2×10^−5^) and Gene Expression (smallest *p* = 5.6×10^−5^). Also, among others, the functional category Neurological Disease (smallest *p* = 2.6×10^−4^) was significantly enriched, containing the genes *CCL5*, *PRKCQ*, *PTAFR*, *AKR1B1*, *CD247*, *IL10RA* and *KHSRP*. A complete overview of the Ingenuity results for the Tan module is given in Table S3. As a caveat, we mention that results from pathway analyses need to be interpreted with some caution since enrichment results can be biased towards well-defined pathways and larger pathways [32]. Therefore, the Ingenuity analysis in this study is primarily used as an explorative tool for examining the gene content of the modules of interest.

### Genome Wide Association Results for Genes in the Tan Schizophrenia Module

We also evaluated the enrichment of the modules with respect to disease related SNPs from a previously published schizophrenia genome wise association study. (1,378 controls and 1,351 cases, NCBI dbGaP analysis accession: pha002857.v1.p1). 115 of the 129 genes in the Tan module were represented by 1,714 SNPs. Visual inspection of the Quantile-Quantile plots (Supporting Information S1) suggests enrichment of the Tan module for nominally associated (*p*<0.05) SNPs. We used Fishers Exact to test for enrichment of nominally associated SNPs against an expected proportion of 5%. We found a significant enrichment of SNPs with *p*<0.05 (Fishers Exact *p* = 1.5×10^−5^, 151 out of 1714 SNPs). Because one gene is represented by multiple SNPs and multiple significant SNPs in one gene could bias the test, we next took the lowest *p*-value per gene (Table S1). Enrichment was significant (Fishers Exact *p* = 2.7×10^−7^, 36 out of 115 SNPs with *p*<0.05).

### Validation of Differential Gene Expression

Real-time quantitative PCR (qPCR) experiments were performed in order to validate expression changes of the number one hub gene of the Tan schizophrenia module, *ABCF1* as well as the gene with the highest log fold change in this module, *GZMH*. We used 165 of the same samples as were used for array hybridization for qPCR validation; for a subset of subjects (n = 34) there was no RNA left but we had access to a second blood draw from which RNA had been extracted. We applied stringent quality control procedures as described in the methods section. For *ABCF1*, we were able to test differential expression in 97 cases and 83 controls whereas measurements for *GZMH* were more variable, leaving 79 cases and 83 controls for analysis. Since high qPCR ΔCt values represent low expression, we expect an inverse correlation with Illumina expression values. Indeed, residuals of Illumina expression values and qPCR ΔCt values are highly negatively correlated (Spearman’s rank correlation *ABCF1*, *r* = -0.60, *p*<2.2e-16; *GZMH*, *r* = −0.88, *p*<2.2e-16). When only selecting the original samples, correlations are even stronger (Spearman’s rank correlation *ABCF1*, *r* = −0.69, *p*<2.2e-16; *GZMH*, *r* = −0.90, *p*<2.2e-16). Confirming Illumina results; *ABCF1* showed decreased expression in cases versus controls (Wilcoxon Ranks sum test, W = 2636, *p* = 6.7×10^−5^, fold change (2^-ΔΔCt^)  = 0.86). *GZMH* reached nominal significance (Wilcoxon Ranks sum test, W = 2353, *p* = 3.8×10^−2^, fold change (2^-ΔΔCt^)  = 0.84). confirming decreased expression in cases.

## Discussion

In this study we utilize gene expression data to identify genes involved in schizophrenia. We identified 2 co-expression modules (gene networks) that consistently relate to schizophrenia status in two independent data sets. One of these modules (under-expressed in schizophrenia patients) was significantly enriched with brain-expressed genes, leading us to focus on this module. The most central (‘hub’) gene in this network, *ABCF1*, is *cis*-regulated, which means that nearby genetic variation indirectly/directly regulates this schizophrenia-associated gene expression network. Using data from a previously published GWAS [33] the overall genes represented in this network are enriched with SNPs nominally associated with schizophrenia, providing further support that this network has a primary role in schizophrenia susceptibility.

In the first test data set, we identified 12 gene co-expression networks (further called modules) that were associated with schizophrenia disease status, reflecting the large number of individual genes found in the differential expression analysis. By relating modules and intramodular hub genes to disease status and SNPs, our systems genetic approach (WGCNA) alleviates the multiple comparison problem inherent in the data: only 14 modules were tested for association instead of >23,000 genes [25], [34], [35], [36].

To address the concern of expression changes induced by the effects of antipsychotics, we evaluated our modules in a second validation dataset of antipsychotic-free samples and found all 14 modules to be highly preserved between datasets, confirming previous observations that it is possible to infer stable and reproducible modules using whole blood RNA expression profiles [37], [38]. In addition, real-time qPCR experiments validated expression changes in the number one hub gene in the Tan schizophrenia module, *ABCF1*, as well as the gene with the highest log fold change, *GZMH*. Although all modules were found to be preserved, only 2 of the 12 schizophrenia-related modules remained significantly related to disease status in the validation data set of antipsychotic-free samples: the Salmon and Tan module. The fact that 10 of the 12 modules were not associated with schizophrenia in the replication set may reflect large-scale effects of antipsychotic medication on gene expression in blood. This phenomenon warrants further investigation since the majority of schizophrenia patients is readily treated and thus may bias other studies likewise. Since schizophrenia is a severe mental disorder often presenting with acute psychosis, it is a major challenge to recruit patients that have not yet received antipsychotic medication. Other considerations that may affect the replication may be the relative small sample size of the 40 controls and 29 antipsychotic-free cases that was used in the second stage, and the source of sampling with no medicated patients collected in Denmark. Due to limited availability of clinical data on the subjects it was not possible to investigate differences in symptom profile, or other parameters such as age of onset. However, there is no evidence that this has led to a systematic bias or a less representative patient group. Nevertheless, the strong conservation of the modules of co-expressed genes in the replication sample and the consistent finding of two modules associated with schizophrenia provide a strong lead for further study.

As expected we find that many co-expression modules reflect blood cell types. Two modules (Blue and Brown) were enriched for neutrophil markers, the most common leukocyte. The Red module was enriched for lymphocyte markers and the Yellow module for red blood cell size markers. Finally, the Cyan module was enriched for genes related to time of blood draw. We did not find that the disease-associated modules (Tan and Salmon) relate to the studied blood cell markers. Since schizophrenia is a brain-related disorder, the question remains whether whole blood gene expression profiles can be informative for analysis. We therefore tested the two significant modules for enrichment of brain-expressed genes, with the assumption that if a module is enriched, this should prioritize our efforts. Interestingly, out of all of the observed modules, only the Tan module is significantly enriched for brain-expressed genes. For this reason we consider the Tan module the most likely to contain genes underlying neuropsychiatric disease. Since we do not have blood and brain samples available from the same individuals, we cannot formally correlate the expression values in these two tissue types.

The gene content and connections for the Tan schizophrenia module are visually represented in Figure 2. Besides categories related to hematological function, the Tan schizophrenia module was also enriched for the Neurological Disease category (*CCL5*, *PRKCQ*, *PTAFR*, *AKR1B1*, *CD247*, *IL10RA* and *KHSRP*). Moreover, this module was found to contain two genes previously suggested to be involved in schizophrenia; namely Catechol-O-methyltransferase (*COMT*) and phosphatidyl-inositol-4-kinase-catalytic-a (*PIK4CA*). *COMT* is involved in the degeneration of endogenous catecholamines as well as in the metabolism of drugs used in many neuropsychiatric diseases. Results from association studies of the common Val158/108Met polymorphism with schizophrenia, however, remain ambiguous [39], [40], [41], [42]. The *PIK4CA* gene is a catalytic enzyme in the phosphatidylinositol (PI) pathway, involved in the regulation of signal transduction, synaptic transmission and possibly of cell shape of neurons or oligodendrocytes. Like *COMT*, this gene has been linked to psychiatric traits associated with the 22 q11.2 deletion syndrome [43], [44], which is in turn characterized by increased prevalence of psychotic symptoms [45], [46].

Previous studies of blood based gene expression of schizophrenia have been modest in size and identified a number of differentially expressed genes but without a consistent pattern [21], [47], [48]. Our relatively large sample size allows us to evaluate previous findings. One study found 123 genes to be differentially expressed in blood, of which 6 showed the same pattern in brain [23]. Our results do not confirm differential expression of these 6 genes. A later study attempting to validate the differential expression of 7 genes from previous studies was only able to confirm the differential expression of *CXCL1* [49]. Our study did not confirm differential expression of *CXCL1*, but of the other 6 genes, *S100A9* (under-expressed in schizophrenia) was found in the Yellow module, one of the modules that is negatively associated to schizophrenia in the medicated dataset.

A larger sample of 49 Japanese antipsychotic-free patients and 52 controls resulted in 792 differentially expressed probes in whole blood. A supervised artificial neural network analysis identified 14 of these as a predictor set for diagnosis with an accuracy of 91.2% [50]. Of the 8 known genes in this list, *PGRMC1* was located in the Blue module, confirming over-expression in schizophrenia. The largest Caucasian blood-based study of schizophrenia consists of 32 untreated patients and 32 matched controls found 180 differentially expressed genes [24]. Of the under expressed genes (97 known genes) we find 17 genes in modules negatively related medicated dataset. Of the over expressed genes (79 known genes), 10 are located in modules positively related to disease status in the medicated dataset. Gene expression analysis in whole blood has been used to investigate the association of psychosis with the ubiquitin proteasome system [51], [52] and a top list of 31 genes [53]. We could only confirm down-regulation of *TCF4* (located in the Red module). The limited overlap in findings characterized gene expression studies of schizophrenia so far both in blood and brain. Although our study confirms a subset of genes found in previous studies, none of them are located in the Tan schizophrenia module. This may result from our large sample size and the fact that we had access to an independent antipsychotic-free sample set to validate our results. Of the genes in the Tan schizophrenia module, 18 genes (*YARS*, *AARS*, *MAGED1*, *HSP90AB1*, *AKR1B1*, *NUP93*, *SNX17*, *DDOST*, *PSMB2*, *NCALD*, *ACOT7*, *IMP3*, *SARS*, *GLG1*, *COMT*, *MXD4*, *GPR177*, *ARAF)* do overlap with co-expression modules constructed using brain tissue and associated with schizophrenia previously [15]. In addition, three genes (*ALDOC*, *ENO1*, *SDHA*) were found to be differentially expressed in previous microarray studies examining brain (overview in [15]).

Intramodular hub genes in disease related modules have been found to be biologically and clinically interesting genes in several disease applications [54], [55]. The most connected intramodular hub gene in the Tan module is *ABCF1* (ATP-binding cassette, sub-family F (*GCN20*), member 1). This gene is located within the MHC region at chromosome 6p. This gene-dense region is essential to the immune system and contains many human polymorphisms [56]. Common variants from the MHC region have been reported to be associated with schizophrenia [2], [3].

The fact that the expression of hub gene *ABCF1* was found to be *cis*-regulated in a previous study suggests that a key member from the Tan module is regulated by the MHC region and in turn drives other genes in the module [31], [57]. The Tan module also contains a number of other genes within the MHC region: heat shock protein 90 kDa alpha (cytosolic), class B member 1 (*HSP90AB1*); ring finger protein 1 (*RING1*); casein kinase 2, beta polypeptide (*CSNK2B*) and tubulin, beta (*TUBB*).

Our results coincide with the findings of existing GWAS studies for schizophrenia that highlighted involvement of the MHC region in disease susceptibility [2], [3]. While GWAS studies included thousands of subjects, our study in contrast involved a few hundred cases and controls. Moreover, gene expression data highlights individual genes within the MHC region that are differentially expressed in schizophrenia while the association signal observed in the GWAS studies are dealing with extensive patterns of linkage disequilibrium without the ability of pinpointing single candidate genes.

It is striking that the robustly defined (Tan) schizophrenia module was significantly enriched with nominally significant SNPs from a GWAS study. This corroborates the fact that this module is independent of drug effects and represents primary disease effects. Previous studies find enrichment of disease related SNPs in their co-expression modules of interest, implicating that these modules represent causal effects [58], [59]. In addition, this approach highlights the fact that network analyses can be used to reconstruct molecular phenotypes for the identification of the genetic association signal derived from pathways, rather than small effects from individual genes.

Overall we show that gene expression profiling in whole blood provides new insight in the molecular networks that may underlie schizophrenia. By making use of an antipsychotic-free patient sample, we were able to replicate our findings and filter out large-scale medication effects. The Tan schizophrenia module that we identified is enriched with brain-expressed genes In addition; there is enrichment of association signal in GWAS, which further supports causal involvement in disease susceptibility. Moreover the association of genetic variants in the MHC region with the hub genes of this Tan schizophrenia module suggests that recent MHC association findings may increase schizophrenia susceptibility via altered gene expression of regulatory genes in this network. Future studies involving suitable model systems could aim to validate these causal hypotheses.

## Materials and Methods

### Subjects

Participants were recruited from three sources: i) the Department of Psychiatry of the University Medical Center Utrecht (90 controls and 113 cases), ii) Parnassia PsychoMedical Center in the The Netherlands (2 cases) and iii) the Center for Neuropsychiatric Schizophrenia Research, Psychiatric Center Glostrup, Denmark (28 controls and 6 cases). Diagnoses were determined by Standardized Psychiatric interviews either The Comprehensive Assessment of Symptoms and History (CASH) or the Composite international diagnostic interview (CIDI) by trained clinicians. Schizophrenia was defined by a DSM-IV-TR diagnosis of #295.0–295.89, and #298.9. All participants gave written informed consent. This study was approved by Medical Research Ethics Committee (METC) of the University Medical Center Utrecht, The Netherlands (accredited on November 1^st^’, 1999 by ex section16 of the WMO) (collection in both Utrecht and the Hague) and the Committees on Biomedical Research Ethics for the Capital Region of Denmark. Antipsychotic-free patients were not on antipsychotics during the six-month-period prior to blood sampling. Only cases with a DSM IV #295.0–295.89 and #298.9 diagnoses were included to increase clinical homogeneity. Since ethnic heterogeneity and relatedness may affect the distribution of genetic variation and consequently gene expression, we removed non-Caucasian subjects as well as closely related subjects as described below. Questionnaire data on ethnicity and relatedness was available for all subjects. In addition, for 119 of the samples in dataset 1 and 2, SNP data was available on Illumina CytoSNP 330k and ethnicity could be assessed using clustering procedures and multidimensional scaling in Plink [60]. After exclusion based on quality control (6 subjects), ethnicity (15 subjects, >2SD from the mean of first two principal components), diagnosis (7 subjects) and relatedness (2 subjects, piHat  = 0.1), 29 antipsychotic free patients, 92 medicated patients and 118 controls remained. Demographic information for both datasets is given in Table 1.

### Expression Arrays

For isolation and purification of mRNA from whole blood the PAXgene extraction kit (Qiagen) were used for all samples. PAXgene tubes were stored in −20°C and RNA was isolated within 6 months after phlebotomy according to the manufacturer’s instructions including an optional DNase digestion step. Total mRNA was quantified using a ribogreen assay (Invitrogen Quant-it™ Ribogreen). Quality of total RNA was determined using Agilent 2100 Bioanalyzer. A threshold of RNA integrity number (RIN) of 7 was taken for selection of RNA samples. Data was generated in two batches. Genome-wide RNA expression profiling was obtained with the Illumina HumanRef-8 V3 arrays for batch 1 and HumanRef-12 V3 arrays for batch 2 using Illumina’s standard protocol at UCLA Illumina facility. The raw microarray data is MIAME compliant and made available at gene expression omnibus (GEO) under accession GSE38485.

### Expression Data Preprocessing

BeadStudio© software version 3.2.3 was used to extract raw data and generate background-corrected gene expression data. Further pre-processing was done using the Lumi package for R. A variance stabilizing transformation (VST) and robust spline normalization (RSN) were applied to the data according to the Lumi procedure [61]. Genes were then filtered based on detection values generated by BeadStudio©. Expression probes had to reach the detection *p*-value threshold <0.01 in at least one sample. Data was split into two datasets, one containing 92 schizophrenia cases and 78 controls, the second all antipsychotic-free subjects (n = 29) and controls (n = 40). Batch effects resulting from the use of different arrays at different time points were removed using the SampleNetwork R function [62]. Data on the probe level was collapsed to genes for separate datasets prior to analyses. When two probes were available (this is the case for most genes) the probe with the highest maximum expression level was selected. In case of three or more probes per gene, the probe with the highest maximum connectivity was selected. This resulted in 16,707 genes in dataset 1 and 12,704 genes in dataset 2.

### Weighted Gene Co-expression Network Analysis (WGCNA)

To identify co-expression modules, we used WGCNA [25], [36], [63], [64], [65]. A detailed description can be found in Supporting Information S2. In short, a weighted adjacency matrix containing pair-wise connection strengths was constructed by using the soft-thresholding approach (β = 6) on the matrix of pair-wise correlation coefficients. A connectivity measure (*k*) per gene was calculated by summing the connection strengths with other genes. Modules were defined as branches of a hierarchical clustering tree using a dissimilarity measure (1 - topological overlap matrix [36], [66]). Each module is subsequently assigned a color. Preservation of module structure was assessed using the modulePreservation R function [26]. The gene expression profiles of each module were summarized by the module eigengene (defined as the first principal component of the module expression levels). Each module eigengene was regressed on disease status (with age and gender as covariates) using the linear model in the limma R package (FDR 5%) [67].

### Public Brain Expression Data

The brain expression data was retrieved via GEO (GSE8919), and consists of 193 neuropathologically normal human brain samples (frontal and temporal cortex) on Illumina HumanRef6 Expression BeadChips [29]. After quality control (using the SampleNetwork R package [62]) 156 samples were used including 24,354 probes collapsed to 19,880 genes. Of these genes, 7,999 (48%) overlap with the 16,707 well-detected genes on the H-12 array. Expression probes had to reach the threshold of a mean log_2_ expression value >4 across all samples to indicate expression in brain (met by 5,753 of the 7,999 (72%) overlapping genes). Significance of overrepresentation (Bonferroni corrected *p*<0.05) was assessed using Fisher’s exact test for count data (hypergeometric distribution).

### Testing Significance of Module in GWAS

Data from a previous schizophrenia GWAS study (NCBI dbGaP analysis accession: pha002857.v1.p1) was available for 1378 controls and 1351 cases, with 729,454 SNPs genotyped using the Affymetrix 6.0 array. We generated a list of SNPs within a 10 kb region around the genes in the co-expression modules and subsequently extracted the empirical *p*-values from the GWAS results. Significance of overrepresentation was assessed using Fisher’s exact test for count data (hypergeometric distribution).

### Real-time Quantitative PCR (qPCR) Experiments

Sufficient RNA from the original sample was available for 80 cases (65 medicated and 15 antipsychotic-free patients) and 85 controls. For another 8 controls and 26 cases the original sample was depleted, but RNA from a second blood tube was available. Total RNA, isolated as previously described, was reverse transcribed to cDNA using the High-Capacity RNA-to-cDNA Kit (AppliedBiosystems) according to manufacturer’s protocol. Following standard protocols, real-time PCR was executed using TaqMan gene expression assays (AppliedBiosystems) for *GZMH* (Hs00277212_m1), *ABCF1* (Hs00153703_m1) and *GAPDH* (Hs03929097_g1) and analyzed on an ABI Prism 7900 System. For all genes, absolute quantities were obtained by running all samples in triplicates. Samples were excluded when raw Ct>35 or SD>0.5 per triplicate. In addition, measurements were excluded when average triplicate Ct values were outside ±2SD per plate for each gene. Ct values were normalized against *GAPDH* expression (ΔCt). Samples were excluded when ΔCt outside ±2SD. Since Illumina expression values were initially subidivided into two datasets, residuals were calculated for a model including dataset as well as age and gender. Residuals for ΔCt values were calculated for a model including pcr plate, age and gender. All residuals were generated using the regression function in the Limma package for R [67]. Differential expression between schizophrenia cases and healthy controls was tested by the Wilcoxon Ranks sum test using the MASS package for R [68].

## Supporting Information

Figure S1 **Preservation of modules between datasets.** A co-expression network for dataset 2 (29 antipsychotic-free and 40 controls) was constructed, using module assignments of dataset 1. The preservation Z-statistic is larger than 2 for all modules, indicating significant module preservation between datasets.
(DOC)

Table S1 **Results of genome-wide WGCNA.** This table contains counts per module for WGCNA performed on genome-wide data (16,707) as compared to analysis performed on the 5,000 most variable genes. The first two columns indicate the modules generated on genome-wide data and the number of genes they contain (highlighted in blue). Horizontally, the first two rows contain the results from the 5,000 most variable genes and their gene counts (highlighted in blue). Color assignments depends on the size of the modules, therefore this is not the same. However, the number of overlapping genes indicates similar grouping in modules. Most of the genes not included in the 5,000 most varying genes are located in the grey ‘noise’ module. The lightgreen module with *ABCF1* as a hub gene contains most genes from the original Tan module. The directionality of the association with schizophrenia disease status remains the same, as is the case for the other modules (module trait correlations and p-values are given).
(XLS)

Table S2 **Module content of Tan module.** Table contains genes in Tan module. Gene symbol, chromosome, k-within statistics, brain-expressed status and log fold changes are given. Expression data is transformed (variance stabilizing method) and normalized (robust spline) yielding values comparable to log_2_ values. Finally, LOD scores and heritability of *cis*-regulated genes identified in a previous study [31] are given.
(XLS)

Table S3 **Ingenuity results for Tan module.** Ingenuity pathway results for genes in the Tan module.
(XLS)

Supporting Information S1 **Q-Q plots for Tan module.** For 115 out of the 129 genes in the Tan module, data from a previous schizophrenia GWAS study was available. Q-Q plots are given for the SNPs in
(DOC)

Supporting Information S2 **Weighted gene co-expression network analysis.** A detailed description of the WGCNA method used in this study.
(DOC)
