# Supplementary figures and images for: A Gene Co-Expression Network in Whole Blood of Schizophrenia Patients Is Independent of Antipsychotic-Use and Enriched for Brain-Expressed Genes
Source: PLoS One. 2012 Jun 27;7(6):e39498. doi: 10.1371/journal.pone.0039498 (PMC3384650; doi:10.1371/journal.pone.0039498)

**A) Tan Module** (1714 SNPs available for 115 of 129 genes)

**B) Genome wide analysis** (729 454 SNPs)


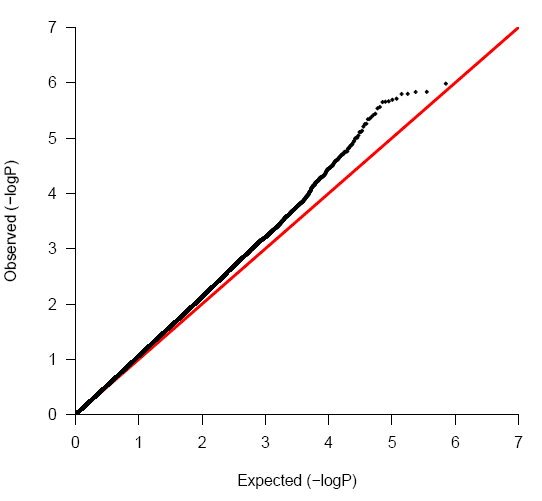

Supplement: Supporting Information S1 — Q-Q plots for Tan module. For 115 out of the 129 genes in the Tan module, data from a previous schizophrenia GWAS study was available. Q-Q plots are given for the SNPs in (DOC) [file pone.0039498.s005.doc]
