# Supplementary material for: A Gene Co-Expression Network in Whole Blood of Schizophrenia Patients Is Independent of Antipsychotic-Use and Enriched for Brain-Expressed Genes
Source: PLoS One. 2012 Jun 27;7(6):e39498. doi: 10.1371/journal.pone.0039498 (PMC3384650; doi:10.1371/journal.pone.0039498)
Supplement: Supporting Information S2 — Weighted gene co-expression network analysis. A detailed description of the WGCNA method used in this study. (DOC) [file pone.0039498.s006.doc]

**Weighted gene co-expression network analysis**

Weighted co-expression networks based on the matrix of pairwise Pearson correlation coefficients were constructed using the soft thresholding approach with a β=6. This method entails raising each correlation to a fixed power by the criterion described by Zhang and Horvath ^30^. Soft thresholding resulted in a 5,000 x 5,000 dimensional weighted adjacency matrix containing pairwise connection strengths. A connectivity measure (*k*) per gene was calculated for every gene by summing the connection strengths with other network genes.

We define co-expression modules as branches of a hierarchical clustering tree. Specifically, we used average linkage hierarchical clustering with a dissimilarity measure derived from the topological overlap matrix to define a cluster tree. This topological overlap measure is calculated based on the number of shared neighbors ^30, 31^. A dendrogram is produced by hierarchical clustering of 1 minus the topological overlap; branches of the tree are cut using a dynamic tree cut algorithm to define modules. For branch cutting (module detection) we used the dynamic branch-cutting algorithm implemented in the dynamicTreeCut and WGCNA (weighted gene co-expression network analysis) R library ^23, 24^. Each module (or branch) is subsequently assigned a unique color label which is visualized in the color band underneath the cluster tree. Connectivity, defined above, can be used to identify ‘hub’ genes that are thought to drive the other genes in a given module. Preservation of module structure was assessed using a set algorithm (Tutorials on module preservation may be found at <http://www.genetics.ucla.edu/labs/horvath/CoexpressionNetwork/ModulePreservation/Tutorials/>)

To define a representative module expression profile for each module, we summarized the (standardized) gene expression profiles of the module by their first principal component. This statistic is referred to as the module eigengene. The module eigengene can be thought of as an average gene expression value for all genes in a module per sample. They were tested for association with disease status (with age and gender as covariates) using the linear model in the limma R package ^32^. An FDR correction level of 5% was used to assess significance. The importance of individual genes could then be assessed by looking at both gene significance and connectivity measures in the whole network and within modules.
