# Supplementary material for: A Gene Co-Expression Network in Whole Blood of Schizophrenia Patients Is Independent of Antipsychotic-Use and Enriched for Brain-Expressed Genes
Source: PLoS One. 2012 Jun 27;7(6):e39498. doi: 10.1371/journal.pone.0039498 (PMC3384650; doi:10.1371/journal.pone.0039498)
Supplement: Supplementary file 6 [file pone.0039498.s001.doc]

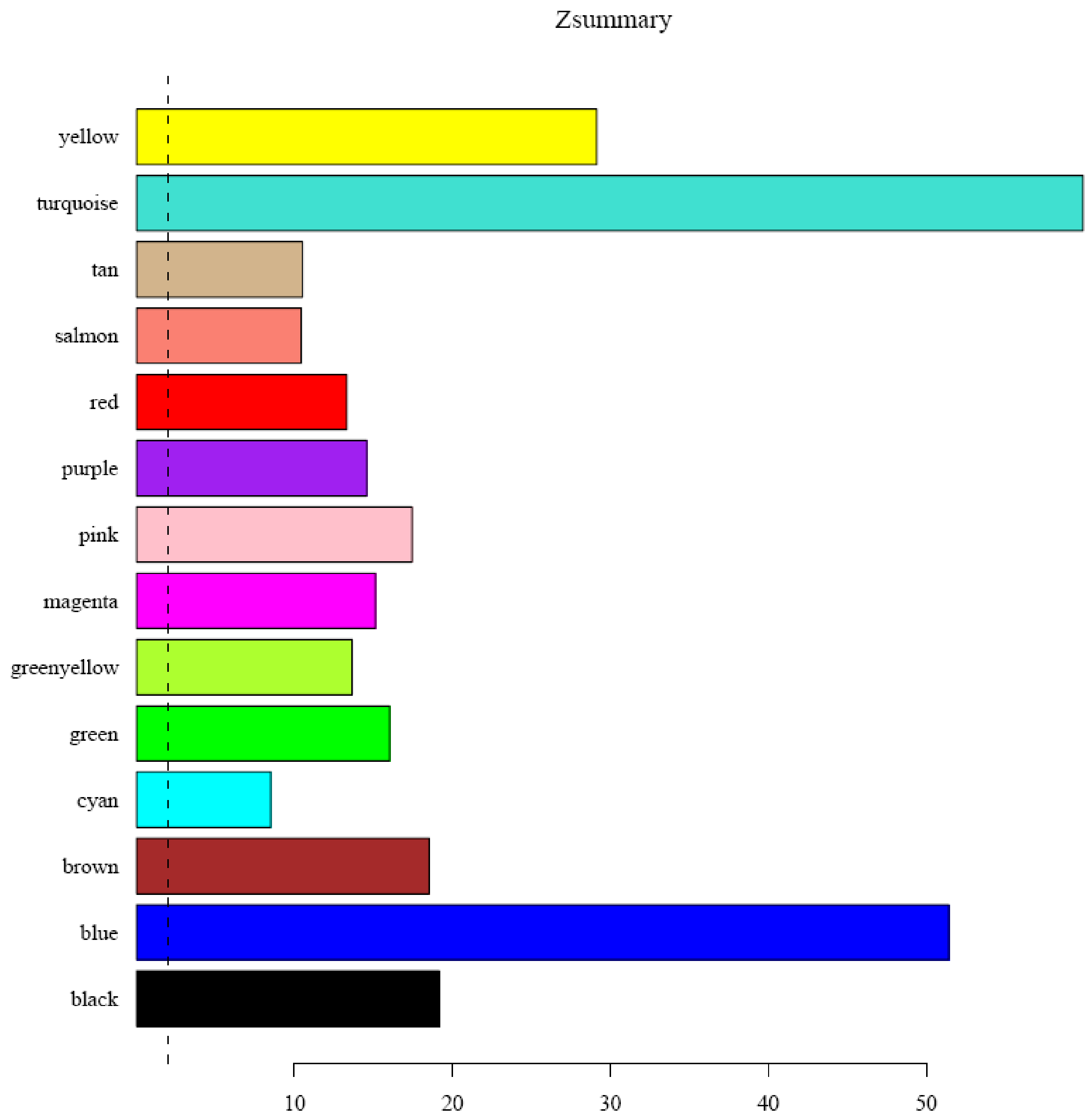
Zsummary
yellow
turquoise
tan
salmon
red
purple
pink
magenta
greenyellow
green
cyan
brown
blue
black
10
20
30
40
50
